# Neck of Femur Fracture among Senior Citizen Managed with Hemiarthroplasty in a Tertiary Care Center in Nepal: An Observational Study

**DOI:** 10.31729/jnma.8844

**Published:** 2024-12-31

**Authors:** Bijay Poudel, Bhaskar Raj Karki, Rohit Shrestha, Ashkal Basi, Sushant Kumar Khadka, Aashutosh Chaudhary, Amartya Dahal, Shreejana Pandey, Darshan Gopal Dhoju

**Affiliations:** 1 Department of Orthopedics and Traumatology, Dhulikhel Hospital, Kavre, Nepal; 2 Kathmandu University School of Medical Sciences, Dhulikhel, Kavre, Nepal; 3 Blood and Multispeciality Hospital, Jawalakhel, Kathmandu, Nepal

**Keywords:** *aged*, *hemiarthroplasty*, *hip fractures*, *modified Harris hip Score*, *neck of femur fracture*

## Abstract

**Introduction:**

Hemiarthroplasty is recognized as a prominent therapeutic option for neck of femur fracture without preexisting acetabular pathology; however, there are controversies in the scientific literature regarding this. The study aims to analyze epidemiology of patients sustaining NoF fracture and the functional outcomes following hemiarthroplasty.

**Methods:**

This is a observational cross-section study in a single tertiary care center of Nepal. Senior citizens, 60 years or above patients, with neck of femur fracture who underwent hemiarthroplasty from January 2017 to December 2022 were included in the study. Patient related data were retrieved from the medical record department of the hospital. A semi-structured proforma was designed which included patient demographic information, operative details and follow up evaluation using Modified Harris Hip Score. Ethical approval was obtained from the Institutional Review Committee (Reference number: 34/24).

**Results:**

The overall mean Modified Harris Hip score for patients who received hemiarthroplasty for neck of femur fracture from January 2017 to December 2022 was 76.36, with 4 (9.30%) achieving excellent results, 19 (41.19%) good, 14 (32.56%) fair, and 6 (13.95%) poor outcomes.

**Conclusions:**

Hemiarthroplasty emerges as a valuable treatment for femoral neck fractures in senior citizens, aged 60 and above, providing good functional results.

## INTRODUCTION

Neck of femur (NoF) fracture is one of the common fractures in elderly population mostly due to trivial trauma.^1^ Early stabilization of fracture and then mobilization is the key to successful management, however this is challenging in most of patients as majority of them have different comorbid conditions and background osteoporosis.^2^ Non-operative management is reserved only for non-ambulatory and severely ill patients.^3^ Operative management is the main stay of treatment, which primarily consists of arthroplasty in senior citizens, that allows early weight bearing mobilization, avoiding complication associated with recumbency.^4^ Hip hemiarthroplasty is recognized as a prominent therapeutic option for

NoF fracture without preexisting acetabular (hip) pathology; however, there are lacunae in the scientific literatures regarding hemiarthroplasty; for example: type of prosthesis, surgical techniques and functional outcome status.^5^

This study mainly aims to analyze epidemiology of patient sustaining NoF fracture and the functional outcomes of patients who were managed with hemiarthroplasty of hip in a tertiary care hospital in Nepal.

## METHODS

We have conducted an observational cross-section study in a single tertiary care center of Nepal after receiving the ethical clearance from the Institutional Review Committee of Kathmandu University School of Medical Sciences (Reference number 34/24).

Senior citizens, aged 60 years or above patients, with NoF fracture who underwent hemiarthroplasty from January 2017 to December 2022 and available for final follow up evaluation at out-patient department, as of December 2023, were included in the study. Polytrauma cases, pathological fracture secondary to neoplastic lesions, hemiarthroplasty done for intertrochanteric fracture, neck of femur managed with other modes of treatment (for example osteosynthesis with cannulated screws and dynamic hip screws) and those patients not available for final follow-up were excluded from study. The study included all patients who matched the inclusion criteria and were not subject to any exclusion criteria. A semi-structured proforma was designed, which included patient demographic information, operative details and follow-up evaluation using Modified Harris Hip Score.^6^ Eight sections are rated including pain, distance walked, ability to wear socks/shoes, public transportation, support needed, limping, ability to climb stairs, and sitting. All the sections are given certain points. Absence of deformity and range of motion are assessed based on the physical examination of the patient. The score ranges from 0 to 100. Scores grouped as follows: 90 to 100 as excellent score, 80 to 89 as good score, 70 to 79 as fair score, and 0 to 69 as poor score. During the patient evaluation and examination, informed consent was obtained from both the patients and their relatives, while gathering information pertaining to the purpose of the study. Demographic and other patient-related data were retrieved from the medical record department of the hospital.

Statistical analysis was done with SPSS Statistics for Windows, version 22.0 (SPSS Inc., Chicago, Ill., USA). The results of the analysis are presented using tables and figures to enhance visual representation and understanding.

## RESULTS

**Figure 1:**
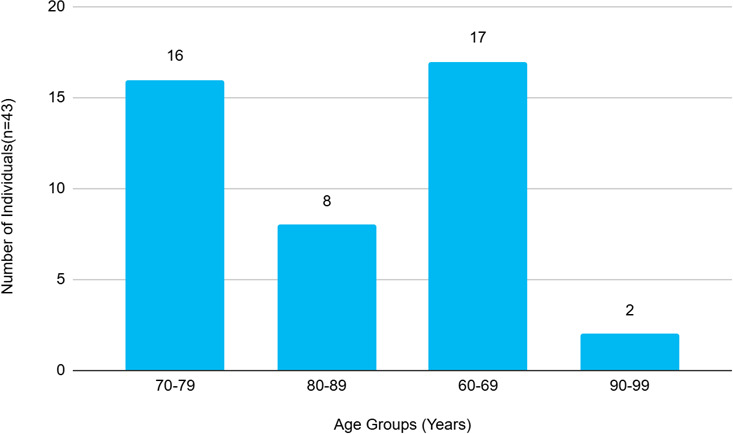
Patients in different age group (n=43).

There were 43 cases that were available for final follow-up evaluation and hence were included in the study. This study comprised 20 (46.51%) male and 23 (53.49%) female, with a male-to-female ratio of 0.9:1. The age range was 60 to 96 years, with a mean age of 73.21± 9.06 years (Figure 1). There were 29 (67.43%) cases from Kavre district, 4 (9.30%) from Sinduli , 3 (6.98%) from Bhaktapur, 3 (6.98%) from Sindhupalchowk, 2 (4.65%) from Ramechhap, 1 (2.33%) from Dolakha and Dhanusha each.

Right hips were involved in 21 (48.84%) cases and left side were involved in 22 (51.16%) cases, bilateral involvement was absent. Cemented thompson prostheses were used in 33 (76.74%) cases, followed by Austin Moore and Bipolar prostheses with 5 (11.63%) cases each. Among these, cement was used in all Thompson cases and one bipolar case, while Austin Moore and two bipolar cases were cementless.

Anesthesia techniques varied, with combined spinal and epidural anesthesia utilized in 34 (79.07%) cases, general anesthesia with endotracheal intubation in six (13.95%) cases, spinal anesthesia alone in three (6.98%) cases. One patient (2.33%) needed conversion from spinal to general anesthesia due to early wear off of spinal anesthesia and was considered in general anesthesia catagory (Table 1).

The median Modified Harris Hip Score for patients operated within six years was 79 (IQR: 74-86) with four patients (9.30%) achieving excellent results, 19 patients (44.19%) good, 14 patients (32.56%) fair, and six (13.95%) patients poor outcomes. Twenty-three (53.49%) patients had one or more comorbid conditions, with hypertension eight (18.60%) and chronic obstructive pulmonary disease (COPD) seven (16.28%) being the most prevalent, followed by diabetes three (6.98%), severe anemia three (6.98%), liver disease one (2.33%), and others one (2.33%). Among these 23 patients, four of them had a variety of comorbidities. Complications related to the operative management included hospital-acquired pneumonia in three (6.98%) patients, superficial wound infection in two (4.65%) patients and heterotopic ossification in one (2.33%) patient, however cementing related per operative problem was not encountered in our series.

**Table 1:** Details on patients and operation parameters (n=43)

Parameters	n(%)
**Gender**	
Male	20 (46.51)
Female	23 (53.49)
**Side**	
Right	21 (48.84)
Left	22 (51.16)
**Types of prosthesis**	
Thompson	33 (76.74)
Austin Moore	5 (11.63)
Bipolar	5 (11.63)
**Cement Use**	
Cemented	34 (79.07)
Uncemented	9 (20.93)
**Anesthesia**	
Combined spinal and epidural	34 (79.07)
GA	6 (13.95)
Spinal	3 (6.98)

## DISCUSSION

Femoral neck fractures represent a significant burden in the elderly population, with increasing prevalence corresponding to rising life expectancies. This study demonstrates that hemiarthroplasty is a viable treatment option for such fractures, particularly in the elderly, despite the complicating factors of comorbid conditions.

In our study, the mean age of patients undergoing hemiarthroplasty was 73.21±9.06 years. This finding bears resemblance to the study conducted by Deepak Jain et al., which reported a mean age of 70.7 years.^7^ This congruence in age distribution suggests potential similarities in the demographic profiles and life expectancies of the Indian and Nepalese populations studied. Conversely, the study by Martyn JP et al in the United Kingdom documented a higher mean age of 80.8 years, potentially indicative of the greater life expectancy prevalent in that geographic region.^8^

Female predominance in our study 23 (53.49%) may be attributed to the higher incidence of osteoporosis and decreased weakened bone mass post-menopause. This ratio is similar to findings by Patel et al. (M:F ratio 1:1.15).^9^

The predominance of cases from Kavre district (67.4%) may reflect the geographic location of the study center followed by neighboring districts Sindhuli, Bhaktapur and Sindhupalchowk. Thompson cemented prosthesis was the most frequently used 76.64%, followed by Austin Moore 11.63% and Bipolar 11.63% implants. While operations in this population are often associated with complications, our study found uneventful outcomes with cement usage in all cases. Advanced anesthesia techniques have likely contributed to improved procedural safety. Conflicting evidence exists regarding cardiovascular risks (eg. hypotension, arrhythmias and cardiac arrests) during cement insertion and mortality rates.^10^ Furthermore, the ongoing discussion regarding the superiority of cemented versus uncemented prostheses and unipolar versus bipolar designs remains unsettled. Some studies indicate a higher incidence of periprosthetic fracture in uncemented prostheses, while no distinct advantages of bipolar prostheses have been identified, especially in elderly populations. Cost considerations may favor unipolar prostheses, especially in developing countries like ours.^11,12^

Anesthesia selection favored combined spinal and epidural anesthesia 79.06%, aligning with study by Chowdary AR et al. indicating its benefits in reducing systemic complications compared to general anesthesia, particularly in patients with multiple comorbidities.^13^ In our study, the mean Modified Harris Hip Score was determined to be 76.36, ranging from 33 to 91. Study conducted by Muhammad Siraj et al. shows excellent in one (2.33%) patient, good in 14 (32.56%) patients, fair in 17 (39.53%) patients and poor in eight patients (18.6%).^14^

Comparatively, a study conducted by Mouzopoulos et al. in subcapital hip fractures, reported Harris Hip Scores of 77.9 at one year and 79.5 at four years of follow-up.^15^ Similarly, Figved et al. in their investigation of cemented and uncemented bipolar prostheses, observed mean Harris Hip Scores of 78.9 and 79.8, respectively, at 12 months post-operation.^16^ These comparative findings shed light on the functional recovery following hemiarthroplasty, contributing to understanding of post-operative outcomes and clinical decision-making in the management of femoral neck fractures.

Our study's outcomes are comparable to these investigations, suggesting similar functional recovery trajectories following hemiarthroplasty for femoral neck fractures. Complications reported by Matthew et al. encompassed periprosthetic fracture, dislocation, infections, deep vein thrombosis (DVT), and perioperative mortality.^17^ Notably, none of our cases experienced periprosthetic fracture or dislocation. However, we encountered complications like hospital-acquired pneumonia and superficial surgical site infection; those were effectively managed with antibiotics, dressings, and physiotherapy as appropriate. Case with heterotrophic ossification was managed conservatively with rest and analgesics; surgical excision was not attempted.

A single-center study and the study conducted among patients available for final follow-up visit within six years of procedure are the limitations of this study. Further multicenter prospective studies with larger populations and longer completed follow-ups could be a future prospect.

## CONCLUSIONS

Hemiarthroplasty emerges as an effective treatment for femoral neck fractures in senior citizens, aged 60 and above providing good functional results. The functional outcome among the study population in the current study was found to be comparable to the other published literatures.
